# Vitamin E-Bonded Membranes Do Not Influence Markers of Oxidative Stress in Hemodialysis Patients with Homozygous Glutathione Transferase M1 Gene Deletion

**DOI:** 10.3390/toxins12060352

**Published:** 2020-05-27

**Authors:** Petar Djuric, Sonja Suvakov, Tatjana Simic, Dragana Markovic, Djurdja Jerotic, Aleksandar Jankovic, Ana Bulatovic, Jelena Tosic Dragovic, Tatjana Damjanovic, Jelena Marinkovic, Radomir Naumovic, Nada Dimkovic

**Affiliations:** 1 Clinical Department for Renal Diseases, Zvezdara University Medical Center, 11000 Belgrade, Serbia; dra.markoviic@gmail.com (D.M.); sashajan223@gmail.com (A.J.); ana.milenic@gmail.com (A.B.); jln_tosic@yahoo.com (J.T.D.); damjanovictatjana1@gmail.com (T.D.); radomirnaumovic450@gmail.com (R.N.); dim@eunet.rs (N.D.); 2 Institute of Medical and Clinical Biochemistry, 11000 Belgrade, Serbia; sonja.suvakov@gmail.com (S.S.); tatjana.simic@med.bg.ac.rs (T.S.); djurdja1.jovanovic@gmail.com (D.J.); 3 Faculty of Medicine, University of Belgrade, 11000 Belgrade, Serbia; 4 Institute of Medical Statistics and Informatics, Faculty of Medicine, University of Belgrade, 11000 Belgrade, Serbia; marinkovic.j@gmail.com

**Keywords:** hemodialysis, oxidative stress, inflammation, vitamin E-bonded membranes

## Abstract

Background: Increased oxidative stress is a hallmark of end-stage renal disease. Hemodialysis (HD) patients lacking glutathione transferase M1 (GSTM1) enzyme activity exhibit enhanced oxidative DNA damage and higher mortality rate than those with active GSTM1 enzyme. To our knowledge, this is the first study to use the vitamin E-bonded membranes (VEM) in patients with homozygous *GSTM1* gene deletion, and we aimed to determine the effect of VEM on oxidative and inflammatory status in HD patients with homozygous *GSTM1* gene deletion. Methods: *GSTM1* genotypes were determined by polymerase chain reaction (PCR) in 170 chronic HD patients. Those with *GSTM1-null* genotype were randomized and 80 were included in the study. Forty of them were dialyzed for three months with VEM, while the other forty were dialyzed with high-flux same-surface polysulfone dialyzers. Markers of protein and lipid oxidative damage and inflammation (thiol groups, malondialdehyde (MDA), Interleukin-6 (IL-6)), together with plasma antioxidant activity (glutathione peroxidase (GPX), superoxide dismutase (SOD)) were determined. Results: Seventy-five patients finished the study. There were no differences at baseline in markers of protein and lipid oxidative damage, inflammation and plasma antioxidant activity. After three months of therapy, GPX, MDA, and thiol groups increased significantly in both groups, but without statistical significance between groups. SOD and C reactive protein (CRP) did not change significantly during the three-month period. IL-6 increased in the control group, and at the same time, decreased in the VEM group, but without statistical significance. Hemoglobin (Hb) value, red blood cells, erythropoiesis resistance index (ERI), serum ferritin and iron did not change significantly within or between groups. Regarding other laboratory parameters, proteins, albumins, triglycerides, serum phosphorus, serum bicarbonate and Kt/V showed significant improvements within groups but with no significant difference between groups. Conclusions: Our data shows that therapy with VEM over three months had no benefit over standard polysulfone membrane in decreasing by-products of oxidative stress and inflammation in dialysis patients lacking GSTM1 enzyme activity.

## 1. Introduction

Patients on maintenance hemodialysis (HD) are constantly exposed to a state of excessive oxidative stress [1]. There are multiple definitions of oxidative stress: one may be that oxidative stress is a signal which induces oxidative reaction and/or affects redox balance, resulting in either stimulation of defense capacity or induction of deleterious damage. It is now clear that reactive oxygen species (ROS) and related species are capable of exerting positive stress, eustress, as well as deleterious effects, distress [2]. HD has been shown to increase the oxidative stress, with ROS being generated on the surface of dialyzer membranes by activated polymorphonuclear leucocytes [3]. Both increased free radicals production and down regulated antioxidant enzymes activities thus contribute to protein, lipid and DNA oxidative damage and by-products accumulation [4,5,6,7]. Also, reduced erythrocyte superoxide dismutase (SOD) and glutathione peroxidase (GPX) activity have been reported in patients with end-stage renal disease (ESRD) [8]. The significant by-products of protein oxidative damage are manifested by decreased thiol (SH-) group content, and loss of thiols has been described in patients with chronic kidney failure [9]. Lipids are also affected by oxidative damage, and determination of the extent of lipid peroxidation includes measurement of malondialdehyde (MDA). It has been demonstrated that plasma MDA values were elevated in HD patients, particularly in those with cardiovascular complications [10].

Members of the glutathione transferase (GST) enzyme superfamily are able to detoxify accumulated uremic toxins in HD patients and possess strong antioxidant activity towards ROS and peroxides [9]. Approximately half of the population lacks GSTM1 enzyme activity, due to a homozygous deletion of the glutathione transferase M1 (*GSTM1*) gene [11]. HD patients lacking GSTM1 activity exhibit enhanced oxidative DNA damage and higher mortality rate than those with an active GSTM1 enzyme [6]. A recent study showed that *GSTM1-null* genotype is a risk factor for general and CV mortality in the HD population [12].

The use of vitamin E-bonded membranes (VEM) is considered to be a way to reduce oxidative stress. This strategy is based on the fact that vitamin E functions as a strong hydrophobic cleaner which provides protection to plasma lipids and lipid peroxidation of cell membranes [11]. Some studies suggest that biocompatible VEM may remove ROS on time and lead to suppression of polymorphonuclear burst. Furthermore, previous studies have shown that the use of VEM either decreased, in some capacity, markers of inflammation or oxidative stress [10,11,12,13]. Since dialysis patients with homozygous deletion of *GSTM1* have been shown to be more prone to oxidative stress and exhibit poorer cardiovascular survival [6,12], we hypothesized that they would benefit from a VEM dialysis regimen in terms of lower oxidative damage and inflammation. Therefore, we performed the first randomized controlled study in order to determine the effect of VEM on by-products of oxidative stress and inflammation in dialysis patients with homozygous deletion of the *GSTM1 gene.*

## 2. Results

### 2.1. Baseline Characteristics

**Figure 1:**
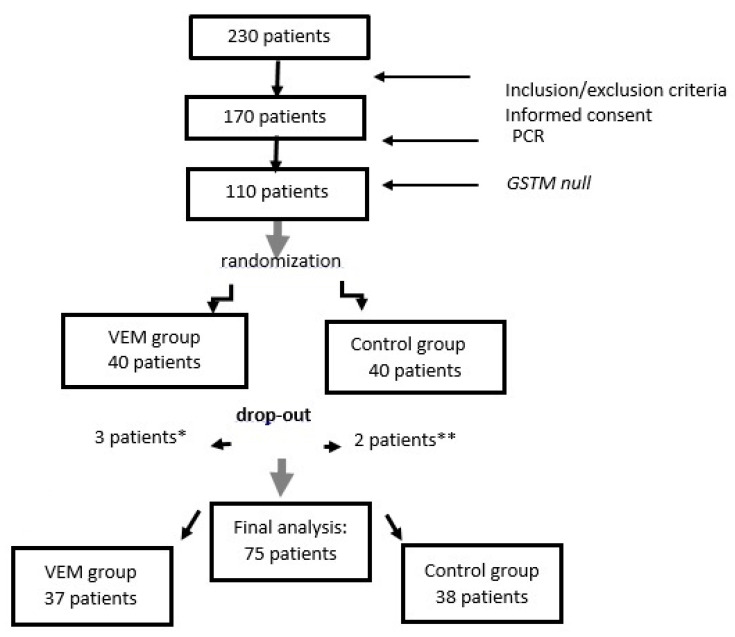
Study flowchart. * stroke, myocardial infarction, one withdrawal. ** hypervolemia and pulmonary edema, chronic obstructive pulmonary disease. VEM, vitamin E-bonded membranes.

Out of 170 tested, 80 patients who had *GSTM1* gene deletion and who fulfilled inclusion/exclusion criteria were randomly selected into two groups: forty patients were randomized to the VEM group and 40 patients to the control group. As shown in Figure 1, three patients from the VEM group dropped out of the trial: one patient died after a stroke, one died after myocardial infarction and one patient withdrew his consent for personal reasons. In the control group, two patients dropped out of the trial—one patient died because of hypervolemia and pulmonary edema, and the other patient died because of chronic obstructive pulmonary disease. Thus, at the end of the trial, 75 patients were available for the final analysis using a per-protocol approach.

Baseline demographic, dialysis and biochemical data of the patients are shown in Table 1. There were no significant differences between the groups regarding any of the parameters assessed. Baseline parameters of anemia, malnutrition, mineral and bone metabolism and inflammation are shown in Table 2. There were no significant differences between the groups regarding any of the parameters assessed, except transferrin saturation and serum-Iron. Patients in the VEM group had better saturation and had higher values of S-Iron.

### 2.2. Primary Endpoint

#### 2.2.1. Markers of Anti-Oxidant Activity

Patients from the control and VEM groups had a significant increase in GPX activity after three months of the study period (intention-to-treat and per-protocol data, Table 3). However, comparison between groups did not show any significant difference (*p* = 0.474). The SOD activity remained unchanged in both tested groups after three months of the study period. 

#### 2.2.2. Markers of Oxidative Damage

After three months, a significant increase of lipid and protein peroxidation products was observed within groups (plasma MDA and Thiol groups), but without a significant difference between groups.

#### 2.2.3. Markers of Inflammation

Inflammatory marker IL-6 showed a modest decrease in patients treated with VEM, whereas those in the control group had increased IL-6 concentration in plasma after the study period (not significant). The CRP remained unchanged in both tested groups.

### 2.3. Secondary Endpoints

Parameters of anemia control and therapy are presented in Table 4. During the three-month period, the total dose of intravenously iron given during the study period was similar (control group versus VEM group, 202 ± 255 mg versus 222 ± 199 mg; *p* = 0.697). Hb value, number of red blood cells, erythropoiesis resistance index (ERI), serum ferritin and serum iron did not change significantly within or between groups. Transferrin saturation decreased in VEM but increased in the control group, and the difference reached statistical significance (*p* = 0.020).

Despite significant improvement in concentrations of proteins, albumins, triglycerides, serum phosphorus, bicarbonate and Kt/V showed significant improvements within groups, no statistical difference was observed for comparisons between VEM and the control group, except for high-density lipoprotein cholesterol (HDL-cholesterol) that was at the edge of statistical significance.

### 2.4. Safety Data

Overall, there were four hospital admissions in the trial subjects (two in the VEM and two in the control group): gastrointestinal bleeding (*n* = 1), polyneuropathy (*n* = 1) and bone fracture (*n* = 2). None of the hospitalizations were considered to be related to the VEM treatment. 

## 3. Discussion

### 3.1. Primary Endpoints

Developments of HD membranes were aimed not only to improve dialysis adequacy but also to improve biocompatibility and to decrease oxidative stress. These advances in HD membrane characteristics have led to an overall improvement of patients’ well-being and quality of life [14]. Vitamin E has been used since the early 1990s as a blood surface modifier of cellulosic first and then synthetic hollow-fiber membranes, with the aim of further improving biocompatibility and eventually providing antioxidant protection to blood cell membranes and circulating lipoproteins [15,16]. Yet, in spite of a wealth of data accruing, there is still no conclusive evidence to prove that there is a clear advantage of these membranes over standard polysulfone membranes [17]. Although patients with GSTM1 deletion are at particular risk for poor outcome due to increased oxidative stress [12], no strategy to prevent this risk has been found to date. Therefore, the availability of VEM has provided a promising opportunity to individualize the treatment and to provide this membrane primarily to the most vulnerable population. Our trial failed to observe significant effects of VEM on the primary study endpoint, i.e., absolute change in the markers of pro-oxidative, antioxidative and inflammatory parameters after three months of therapy.

### 3.2. Markers of Antioxidant Activity

According to presented data, we did not find advantages of VEM membranes over standard polysulfone membranes with regards to markers of antioxidant activity. Namely, after three months of therapy, GPX activity increased significantly in both groups, but without statistical significance between groups. Besides, SOD activity did not change significantly during the three-month period. The increase in GPX activity most probably comes from the increase of the dialysis membrane surface area in both groups during the study (before the study, the average membrane surface was 1.62 and 1.67 m^2^, respectively) but some additional benefits of applying VEM membranes over standard synthetic ones have not been notified by our study. The recent meta-analysis of D’Arrigo et al. [17], which included sixty studies, compared the effect of VEM and conventional HD membranes and concluded that VEM did not affect plasma GPX activity. In agreement with our data, DߣArrigo et al. found that VEM did not produce significant changes in SOD activity. Also, SOD and GPX activity remained unchanged in another study [18].

### 3.3. Markers of Oxidative Damage

VEM did not influence the levels of markers of lipid and protein oxidative damage since concentration of both MDA and thiol groups significantly increased, regardless of the membrane used. In contrast to our data, D´Arrigo et al. found that treatment with VIE decreased MDA level [17]. Besides, a meta-analysis from Yang et al. found a significant reduction of MDA level [19]. Still, it should be noted that in another study, an increase in MDA level was found after three months of therapy with VEM, which is in accordance with the results of our investigation [13]. Data regarding protein oxidative biomarkers, such as thiol group levels after VEM therapy, are still lacking and require further investigation. 

### 3.4. Markers of Inflammation

CRP remained unchanged in both tested groups and IL-6 increased in the control group and at the same time decreased in the VEM group, but the difference between groups did not reach statistical significance. This might be the consequence of marked inter-individual variations in the IL-6 level within both groups. Indeed, the level of IL-6 is not only a marker of blood-membrane contact but also its level is influenced by underlying renal disease, co-morbid conditions, age and therapy applied. Therefore, it is considered more as a non-specific marker of inflammation than the specific marker of membrane pro-oxidative activity. A meta-analysis by D’Arrigo et al. showed data similar to ours that applying VEM resulted in a decrease of the IL-6 level [17]. On the other hand, three randomized controlled trial (RCT) did not confirm such a finding [20,21,22]. Accordingly, a previous meta-analysis demonstrated that the use of VEM significantly reduces IL-6 and CRP levels, hence improving the inflammatory status [20].

### 3.5. Secondary Endpoints

As far as secondary endpoints are concerned, VEM therapy influenced only transferin saturation, which decreased (but within target range) for the same level of iron therapy, erythropoiesis stimulating agents (ESA) dose and ferritin level. This could be explained by better utilization of iron for erythropoiesis in this group of patients. Other parameters of anemia remain the same. Results from the meta-analysis suggested no significant changes in number of erythrocytes, Hb level and average ESA dose by using VEM [17]. Unlike our results, they found a decrease in ERI with the use of VEM, yet this was not recorded in some other RCT studies [23]. Similar to our study, there were no differences in serum iron and ferritin among groups, while there was a significant transferrin saturation (TSAT) decrease. As already outlined by Locatelli’s group [24], the improvement in anemia is usually accompanied with a decrease in IL-6 level, suggesting that the use of VEM may have a beneficial effect on anemia indices. Protection of erythrocyte membranes is mediated by decreased peroxidation and also by reducing the pro-inflammatory cytokines and hepcidin levels that inhibit erythropoiesis and/or alter iron availability for erythropoiesis.

The advantages of VEM over traditional membranes regarding the improvement of nutrition parameters was not found in our study. Although significant improvements were registered in HDL, triglycerides, serum albumin, proteins and Kt/V within groups, no difference between groups was found. Similar results were reported in the above-mentioned meta-analysis [17]. Conversely, LDL and HDL cholesterol levels were decreased in both study groups in another cross-over non-RCT [25], while LDL cholesterol was significantly reduced by VEM in another cross-over RCT [26].

This study has a few limitations. Three months of treatment may not be enough to get insight into the beneficial effects of VEM treatment. Single measurements of inflammatory and oxidative stress parameters may omit variation that may happen (even before and after hemodialysis), thus better insight into the continuous state of inflammation and oxidative stress is missing. By enlarging the number of patients, some values could reach statistical significance. Finally, outcome data are warranted after a longer duration of therapy. However, as the first randomized study conducted over high-risk patients (with GSTM1 deletion), it gives us the impression that the benefits of VEM, one expensive membrane, need to be clearly documented in the future.

## 4. Conclusions

In conclusion, our data shows that therapy with VEM over a three-month period has no benefit over standard polysulfone membrane in decreasing by-products of oxidative stress and inflammation in high-risk dialysis patients. Given the few and controversial results in the literature, new studies in this area are needed.

## 5. Materials and Methods

### 5.1. Patients and Trial Protocol

The trial was single-blind (blind for investigator), randomized, placebo-controlled and it was conducted over 3 months. The study began on 30 July 2018 and the last treatment was completed on 30 October 2018. Out of 230 patients in the Clinical Department of Renal Diseases, Zvezdara University Medical Centre, we recruited 170 patients who fulfilled the inclusion criteria and who were willing to participate in the study. The study was approved by the local Ethical Board (number of authorization of the study by local Ethical Board, 18062018/2018; approved date 18 June 2018.) and all patients signed informed consent after detailed information about the study protocol. Inclusion criteria were: being on chronic thrice weekly HD for more than 3 months, age above 18 years and ability to give informed consent. Exclusion criteria were: pregnancy, involvement in another study, active/chronic inflammation, malignancy, antioxidant and anti-inflammatory therapy.

GSTM1 genotyping was done to 170 patients. Homozygous deletion for the GSTM1 gene was found in 110 patients. According to current National Health Care standards about dialysis membranes, we were allowed to recruit 80 patients into the study due to the membrane cost. Patients were randomly selected in two groups (aided by the program available online at the website (http:/www.graphpad.com/quickcalcs/randomize1.cfm)). Patients in the intervention group (*n* = 40) were treated using VEM (Vitabran E, Asahi Kasei Medical Corporation Limited, Japan, 1.8 m^2^ surface) three times a week for 4 h over the period of three months, while the patients in the control group were treated using a synthetic polysufone membrane of the analogous size for the same period of time (FX80, Fresenius Medical Care, Bad Homburg, Germany, 1.8 m^2^ surface). Dialysis was performed with ultra-pure dialysis fluid. The effect of the therapy was documented by comparing the analyses taken basally (before the study) and after the three-month study period.

The primary endpoint of the trial was the absolute change in the markers of pro-oxidative, antioxidative and inflammatory parameters at the end of the study as compared to baseline. Secondary endpoints included changes of the parameters of anemia and nutrition, all determined as described below. Finally, adverse events were recorded as reported by the patients and treating physicians.

### 5.2. Demographic, Biochemical and Dialysis Data

Patient characteristics were taken from the medical records and included age, sex, dialysis vintage (months), presence of diabetes mellitus and hypertension.

Routine laboratory data were captured as the average of 3 measurements before the start and at the end of the trial. Samples for laboratory analyses were obtained before the HD procedure after a three-day interval.

The adequacy of dialysis was assessed using Kt/V values calculated according to the Daugirdas formula [27]. The erythropoiesis resistance index (ERI) was defined as the weekly ESA dose (IU) divided by the product of the patient’s weight (kg) and the hemoglobin level (g/dL). A conversion ratio of 1:200 was used to convert the darbepoetin dose (μg) to international units (IU) of epoetin as per convention [28].

### 5.3. Glutathione Transferase (GST) Genotyping

Genomic DNA was isolated from whole blood using the QIAGEN QIAmp kit (Qiagen, Inc., Chatsworth, CA, USA).

### 5.4. Determination of GSTM1 Gene Polymorphism

The analysis of GSTM1 gene deletion polymorphism was performed by multiplex polymerase chain reaction (PCR) [29]. PCR reaction mixture was incubated and initially denatured at 94 °C for 3 min. After that, the following steps were performed: denaturation (94 °C, 30 s), annealing (30 s 59 °C) and primer extension (1 min at 72 °C). Final extension lasted for 4 min at 72 °C. PCR products were separated on a 2% agarose gel at 125 V for 20 min and stained with ethidium bromide. The GSTM1 DNA fragments that were amplified were 215 bp in size. The absence of the 215 bp band was indicative for the *GSTM1-null* genotype. The presence of the *GSTM1*-*active* genotype (referent genotype) was confirmed by the band at 215 bp. The assay does not distinguish heterozygous from homozygous reference genotype.

### 5.5. Plasma Separation

Venous blood samples (approximately 5 mL) were collected in standard sterile polystyrene vacuum tubes containing ethylene diamine tetra acetic acid (EDTA) at the beginning of the dialysis session prior to the administration of heparin. For plasma separation, samples were centrifuged at 3600 rpm for 10 min. Plasma samples were aliquoted to avoid frequent thawing/freezing and stored at −80 °C until further usage.

### 5.6. Protein Thiol Groups

Total content of protein thiol groups in plasma was determined according to the method previously described by Jocelyn [30]. This method is based on reactions of thiols with Ellman’s reagent (5,5′-dithiobis-(2-nitrobenzoic acid), or DTNB. Protein thiol groups react with DTNB cleaving the disulfide bond to give 2-nitro-5thiobenzoate (TNB−), which will ionize to the TNB2− dianion in water at neutral and alkaline pH. This TNB2− ion has a yellow color. Molar extinction coefficient for TNB is 13.6 × 103 L⋅mol^−1^⋅cm^−1^ at a 412 nm wavelength. Since the sunlight can reduce DTNB, all reactions were performed in a dark place or protected from sunlight.

### 5.7. Measurement of Malondialdehyde Level

MDA levels were measured using the competitive enzyme immunoassay (Elabscience, Wuhan, China) following the manufacturer’s protocol. The colour change was measured spectrophotometrically at a wavelength of 450 nm. The concentration of MDA was expressed as ng/ml.

### 5.8. Measurement of IL-6 Level

Plasma interleukin-6 (IL-6) concentration was measured using an ELISA kit according to the instruction manual (Human IL-6 (Interleukin 6) ELISA Kit, Reactivity: Human, Detection 96T, Elabcsience).

### 5.9. Determining the Activity of Antioxidant Enzymes

The activity of superoxid dismutase (SOD) and glutathione peroxidase (GPX) in all patients was determined by using spectrophotometric methods. The SOD activity was measured by the method described by Misra and Fridovich [31]. Briefly, the assay is based on the capability of SOD to inhibit spontaneous autoxidation of adrenalin in an alkaline environment (pH = 10.2). The SOD activity is expressed as a percentage of inhibition of adrenaline autoxidation. GPX activity was assessed by monitoring the oxidation of NADPH at 340 nm in the presence of hydrogen peroxide [32].

### 5.10. Statistics

Statistical analyses were performed using the Statistical Package for the Social Sciences SPSS (version 22.0) program. Data were expressed as frequencies or percentages for discrete variables, and mean values with standard deviations for continuous variables. Intention-to-treat and per-protocol data were analyzed. Statistical analyses included exploratory descriptive and analytic statistics. The independent sample *t*-test was used to compare variables with a normal distribution between different groups. In cases where variables did not have a normal distribution, the Mann–Whitney test was used. One-way analysis of variance (ANOVA) with repeated measurements was used to compare two group means, where the participants are the same in each group. This usually occurs when participants are measured multiple times to see changes to an intervention on the data collected at two time points. *p* < 0.05 was considered to be statistically significant.

## Figures and Tables

**Table 1:** General and biochemical characteristics of the patients at baseline.

Variable	Control Group *n* = 40	VEM Group *n*= 40	*p*
Males, %	55	60	0.651
Age, years	65 ± 12	62 ± 11	0.225
Mean Rank (Sum of Ranks)	44.1 (1764)	36.9 (1476)	0.160
Body weight kg (mean ± SD)	69.8 ± 15.4	74.3 ± 14.8	0.182
Mean Rank (Sum of Ranks)	36.1 (1472)	44.2 (1768)	0.427
AVF, No, %	39/97.5	36/90.0	0.210
Dialysis vintage, months	71.5 ± 65.7	66.6 ± 58.7	0.726
Diabetes (main disease or co-morbidity), No, %	11/27.5	12/32.2	0.875
IM (yes) No, %	4/10	2/5	0.675
Stroke (yes) No, %	1/2.5	1/2.5	1.000
HTA (yes) No, %	39/97.5	39/97.5	1.000
ESRD assumed due to (No, %):			0.273
Hypertension	19/47.5	12/30.0	
Diabetes mellitus	7/17.5	6/15.0	
Glomerulonephritis	6/15.0	5/12.5	
ADPKD	4/10.0	10/25.0	
Other	4/10.0	7/17.5	
HDF (yes) No, %	6/15	10/25	0.264
Dialyzer surface, m^2^	1.62 ± 0.27	1.67 ± 0.32	0.407
S-urea, mmol/L	23.1 ± 7.1	23.0 ± 6.0	0.981
S-Creatinine, umol/L	872 ± 211	853 ± 154	0.649
S-Bicarbonate, mmol/L	20.3 ± 2.4	13.0 ± 3.8	0.782
Kt/V	1.32 ± 0.31	1.35 ± 0.30	0.657
Mean Rank (Sum of Ranks)	39.2 (1569)	41.78 (1671)	0.624
Kt/V in target, %	68.4	73.0	0.801
CRP, mg/L	4.57 ± 5.07	5.49 ± 5.29	0.430
CRP in reference range, %	72.5	60.0	0.344

AVF: arteriovenous fistula, MI: myocardial infarction, HTA: arterial hypertension, ESRD: end-stage renal disease, HDF: hemodiafiltration, CRP: C-reactive protein. ADPKD: adult dominant polycystic kidney disease.

**Table 2:** Parameters of anemia, malnutrition, inflammation and mineral/bone metabolism at the baseline.

Parameter	Control Group *N* = 40	VEM Group *N* = 40	*p*
ESA (yes) No, %	31/77.5	32/80.0	0.785
ESA dose, IU/week	6677 ± 3995	5156 ± 3380	0.108
Hb, g/dL	10.5 ± 1.0	10.5 ± 1.0	0.874
Hb in target range, %	92.5	92.5	1.000
ERI	11.3 ± 10.1	7.4 ± 5.6	0.063
Transferrin saturation, %	28 ± 9	34 ± 10	0.014
Ferritin, ng/mL	212 ± 224	258 ± 146	0.281
Albumin, g/L	39.0 ± 2.9	40.1 ± 3.3	0.165
Serum-Iron, umol/L	10.8 ± 3.7	13.0 ± 3.8	0.010
Total proteins, g/L	68.3 ± 5.5	67.6 ± 5.0	0.552
PTH, pg/mL	278 ± 361	290 ± 524	0.905
Total Cholesterol, mmol/L	4.25 ± 0.98	4.54 ± 0.88	0.170
HDL-Cholesterol, mmol/L	1.08 ± 0.41	0.99 ± 0.24	0.260
LDL-Cholesterol, mmol/L	2.38 ± 0.75	2.67 ± 0.79	0.106
Triglycerides, mmol/L	1.83 ± 1.24	2.22 ± 1.22	0.161
S-Ca, mmol/L	2.29 ± 0.24	2.29 ± 0.23	0.925
S-PO4, mmol/L	1.61 ± 0.57	1.8 ± 0.51	0.109

ESA: erythropoiesis stimulating agents, ERI: erythropoietin resistance index, HDL: high-density lipoprotein cholesterol, LDL: low-density lipoprotein cholesterol, Hb: hemoglobin, S-Ca: serum calcium, S-PO4: serum phosphate, iPTH: intact parathormone.

**Table 3:** Markers of plasma antioxidant activity, protein and lipid oxidative damage and inflammation before and after therapy (intention-to-treat and per-protocol data).

Parameter	Control Group		VEM Group			
	Before	After	Before	After	*p*	*p* *
**Markers of anti-oxidant activity**						
GPX, nmol/mg					0.000	0.474
PP data	206.7 ± 67.2	273.7 ± 61.6	203.7 ± 59.1	285.7 ± 78.7		
ITT data	204.6 ± 66.9	273.7 ± 61.6	201.7 ± 60.6	285.7 ± 78.7		
SOD, U × 10^3^/L					0.269	0.607
PP data	55.4 ± 11.7	60.4 ± 22.4	53.8 ± 9.8	55.1 ± 21.2		
ITT data	55.4 ± 11.5	60.4 ± 22.4	53.8 ± 9.5	55.7 ± 21.2		
**Markers of oxidative damage**						
MDA, ng/mL						
PP data	510.9 ± 394.7	69.5 ± 440.7	403.4 ± 218.8	752.1 ± 439.0	0.000	0.446
ITT data	691.5 ± 478.3	69.5 ± 440.7	598.5 ± 425.7	729.9 ± 438.7		
Mean Rank (Sum of Ranks)	32.6 (978)	21.5(430)	29.4(912)	19.5(389)		
Thiol groups, mcmol/L					0.000	0.445
PP data	6.79 ± 1.81	8.33 ± 2.26	7.31 ± 2.58	8.31 ± 2.48		
ITT data	6.69 ± 1.86	8.33 ± 2.26	7.23 ± 2.61	8.31 ± 2.48		
**Markers of inflammation**						
IL-6, ng/mL						
PP data	48.5 ± 40.7	67.6 ± 120.2	34.4 ± 32.4	34.4 ± 32.4	0.543	0.376
ITT data	37.5 ± 34.8	67.6 ± 120.2	34.5 ± 26.3	33.3 ± 31.4		
Mean Rank (Sum of Ranks)	37.2(1412)	26.1(548)	37.9(1363)	23.3(628)		
CRP, mg/L					0.458	0.894
PP data	4.9 ± 5.4	5.5 ± 6.2	5.1 ± 4.8	5.9 ± 7.0		
ITT data	4.6 ± 5.1	5.5 ± 6.2	5.5 ± 5.3	5.9 ± 7.0		

*p*: Within groups *p* *: Between groups. GPX: glutathione peroxidase, SOD: superoxide dismutase, MDA: malondialdehyde, IL-6: Interleukin-6, PP: per-protocol, ITT: intention-to-treat.

**Table 4:** Laboratory parameters and anemia status/therapy before and after the study (intention-to-treat and per-protocol data).

Parameter	Control Group		VEM Group			
	Before	After	Before	After	*p*	*p* *
ESA dose, IU/week					0.427	0.491
PP data	6862 ± 4033	6344 ± 3957	5630 ± 3443	5593 ± 3765		
ITT data	6677 ± 3995	6000 ± 3856	5156 ± 3380	5379 ± 3717		
ESA (yes) No, %					0.540	0.213
PP data	31/81	33/86.8	31/83.8	29/78.4		
ITT data	31/77.5	33/86.8	32/80.0	29/78.4		
Transferrin saturation, %					0.766	0.020
PP data	28.0 ± 8.8	33.0 ± 11.5	33.8 ± 10.1			
ITT data	28.0 ± 9.0	32.8 ± 11.5	34.0 ±10.0	30.4 ± 10.9		
Ferritin, ng/mL					0.262	0.061
PP data	215 ± 230	271 ± 274	245 ± 141	230 ± 165		
ITT data	212 ± 224	272 ± 274	258 ± 146	230 ± 165		
Iron dose, mg	203 ± 256		222 ± 199			0.697
S-Iron, umol/L					0.817	0.087
PP data	10.8 ± 3.8	11.7 ± 4.2	13.1 ± 3.7	11.9 ± 4.1		
ITT data	10.8 ± 3.7	11.7 ± 4.2	13.0 ± 3.8	11.9 ± 4.1		
Hb, g/dL					0.347	0.229
PP data	10.48 ± 0.94	10.17 ± 0.93	10.49 ± 1.06	10.53 ± 1.37		
ITT data	10.50 ± 1.00	10.17 ± 0.93	10.50 ± 1.00	10.53 ± 1.37		
Er, 10^12^/L					0.598	0.053
PP data	3.49 ± 0.35	3.36 ± 0.34	3.42 ± 0.36	3.49 ± 0.51		
ITT data	3.49 ± 0.34	3.36 ± 0.34	3.42 ± 0.40	3.49 ± 0.51		
Hematocrit, %					0.313	0.200
PP data	32.9 ± 2.7	31.8 ± 2.8	32.8 ± 3.5	32.9 ± 4.2		
ITT data	33.0 ± 2.8	31.8 ± 2.8	32.8 ± 3.4	32.9 ± 4.2		
ERI					0.352	0.310
PP data	11.77 ± 10.28	10.67 ± 9.75	8.08 ± 5.78	8.13 ± 6.29		
ITT data	11.30 ± 10.10	10.00 ± 9.35	7.40 ± 5.60	7.87 ± 6.20		
Total protein, g/L					0.048	0.853
PP data	68.3 ± 5.5	69.5 ± 5.7	67.2 ± 5.0	68.2 ± 5.9		
ITT data	68.3 ± 5.5	69.5 ± 5.7	67.6 ± 5.0	68.2 ± 5.9		
Albumin, g/L					0.003	0.421
PP data	39.08 ± 2.96	40.87 ± 4.94	39.97 ± 3.26	41.03 ± 4.29		
ITT data	39.00 ± 2.90	40.87 ± 4.94	40.10 ± 3.30	41.03 ± 4.29		
Uric acid, umol/L					0.069	0.333
PP data	365 ± 67	339 ± 64	346 ± 67	338 ± 71		
ITT data	364 ± 65	339 ± 64	345 ± 65	338 ± 71		
S-Creatinine, umol/L					0.092	0.179
PP data	872 ± 217	819 ± 247	860 ± 158	854 ± 159		
ITT data	872 ± 211	819 ± 247	853 ± 154	854 ± 159		
S-Urea, mmol/L					0.021	0.540
PP data	23.2 ± 7.3	20.1 ± 5.8	22.7 ± 6.0	21.4 ± 6.2		
ITT data	23.1 ± 7.1	20.1 ± 5.8	23.0 ± 6.0	21.4 ± 6.2		
Total Cholesterol, mmol/L					0.361	0.986
PP data	4.23 ± 1.00	4.20 ± 0.91	4.57 ± 0.87	4.50 ± 1.25		
ITT data	4.25 ± 0.98	4.14 ± 0.91	4.54 ± 0.88	4.50 ± 1.25		
HDL-Cholesterol, mmol/L					0.004	0.064
PP data	1.09 ± 0.42	1.11 ± 0.50	0.99 ± 0.25	1.10 ± 0.28		
ITT data	1.08 ± 0.41	1.11 ± 0.50	0.99 ± 0.24	1.10 ± 0.28		
LDL-Cholesterol, mmol/L					0.553	0.641
PP data	2.36 ± 0.76	2.26 ± 0.73	2.68 ± 0.80	2.67 ± 1.05		
ITT data	2.38 ± 0.75	2.26 ± 0.73	2.67 ± 0.79	2.60 ± 1.06		
Triglycerides, mmol/L					0.003	0.102
PP data	1.80 ± 1.27	1.66 ± 1.14	2.28 ± 1.25	1.77 ± 0.74		
ITT data	1.83 ± 1.24	1.66 ± 1.14	2.22 ± 1.22	1.77 ± 0.74		
S-Ca, mmol/L					0.052	0.562
PP data	2.27 ± 0.23	2.31 ± 0.19	2.30 ± 0.23	2.37 ± 0.27		
ITT data	2.29 ± 0.24	2.31 ± 0.19	2.29 ± 0.23	2.37 ± 0.27		
S-PO4, mmol/L					0.000	0.909
PP data	1.61 ± 0.59	1.39 ± 0.51	1.82 ± 0.52	1.59 ± 0.50		
ITT data	1.61 ± 0.57	1.39 ± 0.51	1.8 ± 0.51	1.59 ± 0.50		
Serum bicarbonate, mmol/L					0.009	0.664
PP data	20.32 ± 2.47	21.55 ± 1.48	20.60 ± 4.63	21.49 ± 3.00		
ITT data	20.31 ± 2.40	21.55 ± 1.48	13.01 ± 3.82	21.49 ± 3.00		
Kt/V					0.044	0.567
PP data	1.32 ± 0.29	1.39 ± 0.33	1.35 ± 0.31	1.39 ± 0.25		
ITT data	1.32 ± 0.31	1.39 ± 0.33	1.35 ± 0.30	1.39 ± 0.25		

*p*: Within groups *p* *: Between groups. ESA: erythropoiesis stimulating agents, Hb: hemoglobin, Er: erythrocyte, ERI: erythropoietin resistance index, HDL: high-density lipoprotein cholesterol, LDL: low-density lipoprotein cholesterol, S-Ca: serum calcium, S-PO4: serum phosphate, PP: per-protocol, ITT: intention- to-treat.
